# Persistent presence of outer membrane epitopes during short- and long-term starvation of five *Legionella pneumophila* strains

**DOI:** 10.1186/s12866-018-1220-x

**Published:** 2018-07-17

**Authors:** Barbara Schrammel, Markus Petzold, Sílvia Cervero-Aragó, Regina Sommer, Christian Lück, Alexander Kirschner

**Affiliations:** 1 Institute for Hygiene and Applied Immunology - Water Hygiene, Center for Pathophysiology, Infectiology and Immunology, Medical University of Vienna, Kinderspitalgasse 15, A-1090 Vienna, Austria; 2 Institute for Medical Microbiology and Hygiene, Medical Faculty “Carl Gustav Carus”, University of Technology Dresden, Dresden, Germany; 3 Interuniversity Cooperation Centre for Water and Health, Vienna, Austria

**Keywords:** Viable but nonculturable, *Legionella*, Epitope, Outer membrane, Persistence, Immuno-fluorescence, ELISA

## Abstract

**Background:**

*Legionella pneumophila*, the causative agent of Legionnaire’s disease, may enter a viable but non-culturable (VBNC) state triggered by environmental stress conditions. Specific outer-membrane epitopes of *L. pneumophila* are used in many diagnostic applications and some of them are linked to important virulence-related factors or endotoxins. However, it is not clear how the presence and status of these epitopes are influenced by environmental stress conditions. In this study, changes of outer membrane epitopes for monoclonal antibodies (mAb) from the Dresden panel and the major outer membrane protein MOMP were analysed for five *L. pneumophila* strains during short- and long-term starvation in ultrapure water.

**Results:**

With ELISA and single cell immuno-fluorescence analysis, we could show that for most of the investigated mAb-strain combinations the total number of mAb-stained *Legionella* cells stayed constant for up to 400 days. Especially the epitopes of mAb 3/1, 8/5, 26/1 and 20/1, which are specific for *L. pneumophila* serogroup 1 subtypes, and the mAb 9/1, specific for serogroup 6, showed long-term persistence. For most mAb- stained cells, a high percentage of viable cells was observed at least until 118 days of starvation. At the same time, we observed a reduction of the fluorescence intensity of the stained cells during starvation indicating a loss of epitopes from the cell surface. However, most of the epitopes, including the virulence-associated mAb 3/1 epitope were still present with high fluorescence intensity after 400 days of starvation in up to 50% of the starved *L. pneumophila* population.

**Conclusions:**

The results demonstrate the continuous presence of outer membrane epitopes of *L. pneumophila* during short-term and long-term starvation. Thus, culture-independent mAb-based diagnostic and detection tools, such as immuno-magnetic separation and microarray techniques are applicable for both *L. pneumophila* in the culturable and the VBNC state even after long-term starvation but nevertheless require careful testing before application. However, the mere presence of those epitopes is not necessarily an indication of viability or infectivity.

**Electronic supplementary material:**

The online version of this article (10.1186/s12866-018-1220-x) contains supplementary material, which is available to authorized users.

## Background

*Legionella* is ubiquitously found in fresh-water environments and can grow to high numbers in man-made water-systems at elevated temperatures above 20 °C. Inhalation of *Legionella*-containing aerosols may lead to severe lung infections in humans, called Legionnaire’s disease, with an overall case fatality rate of 8% [1] or to a milder non-fatal form, called Pontiac Fever. In 80% of culture-confirmed Legionnaire’s disease cases *L. pneumophila* serogroup (SG) 1 has been identified as the causative agent. *Legionella* cells usually replicate intracellularly in food vacuoles of their natural hosts, the free-living amoebae. Legionellae and amoebae both inhabit biofilms of natural and engineered water systems, where amoebae graze and take up the bacteria as nutrient source. Legionellae are able to withstand digestion, replicate in the amoebae and finally evade the host cell in high numbers [2]. Similarly, lung macrophages prone to destroy invading microorganisms, accidently serve as host organisms, resulting in severe pneumonia [3].

The components and characteristics of *Legionella*’s cell envelope especially influence the ability of the bacterium to cause Legionnaire’s disease [4]. The major immuno-dominant antigen [5] recognised by patients sera is the lipopolysaccharide (LPS) layer of the outer membrane, which exists in all *Legionella* species and similarly in other gram-negative bacteria. The LPS consists of three different parts, the O-specific antigen, the core region and the lipid A, which is composed of unusually long chain fatty acids. These may be responsible for the weaker endotoxic activity of the molecule in comparison to other gram-negative bacteria [5] and might help to evade the innate immune system [4]. The core-region and the O-specific chain provide various binding sites for monoclonal antibodies (mAbs). Since these regions are highly diverse among strains and serogroups [6], a variety of mAbs are used for serotyping schemes such as the Dresden panel [7].

*L. pneumophila* SG1 strains possessing the mAb-3/1-epitope have often been associated with enhanced virulence, e.g. they were found to be the infectious agent in 66% of all patient samples analysed in a pan-European study [8]. Previously, it was demonstrated that the mAb 3/1 specifically recognizes an 8-O-acetylated saccharide named legionaminic acid, which is the major component of the O-specific antigen part of the LPS [6]. It is proposed, that the high degree of acetylation and thus hydrophobicity of *Legionella* LPS mAb 3/1 positive strains could lead to an enhanced binding to host cells [4] or a better survival in aerosols [6] which are responsible for the transmission of the bacteria. Above all, the LPS plays an additional role in the modulation of intracellular trafficking in the host cell, independently of the Dot/Icm secretion system [4].

The major outer membrane protein (MOMP) consists of 28 kDa subunits and is the most abundant protein in the outer membrane of *L. pneumophila* [9]. MOMP has a porin function and acts as receptor of the innate immune complement system enhancing the phagocytosis by human monocytic cells [10]. Thus, the expression of MOMP constitutes an important virulence factor.

The LPS and outer membrane antigens are widely used for diagnostic purposes. The urinary antigen test is the most frequently used diagnostic tool to identify Legionnaire’s disease [1]. Additionally, the LPS plays an important role in several recently developed *Legionella* detection systems for the surveillance of water systems. Immuno-magnetic separation (IMS) for example becomes a more and more applied technique in water samples with high background microbiota. IMS systems such as the “cell stream” system (rqmicro, Zürich, Switzerland) specifically capture *L. pneumophila* SG1 cells in a water sample by using mAbs bound to magnetic particles; isolated bacteria are then suitable for downstream analysis of culturability, viability and quantification via qPCR or flow cytometry [11–14]. Furthermore, a new microarray technology, the LegioTyper, which consists of mAbs from the Dresden panel as capture-antibodies spotted on a microarray for fast, direct detection and serotyping of *L. pneumophila* in clinical and environmental samples is under development [15]. Moreover, in one study a specific and direct detection assay based on mAbs in combination with viability testing by solid phase cytometry for the analysis of *L. pneumophila* in water systems was established [16].

Legionellae in engineered water systems are often exposed to stress conditions such as nutrient depletion, heat treatment or oxidizing reagents like chlorine. Encountering such conditions, legionellae change gene expression to optimize stress management and consecutively enter a dormant and viable but nonculturable (VBNC) state [17]. Those cells cannot be cultured on routine media anymore, but may retain viability and infectivity for long periods of time [18–21].

Until now, it has never been investigated, whether VBNC *Legionella* cells keep their outer membrane intact or whether parts of the outer membrane are destroyed or rebuilt. It was only shown, for example, that mAb 3/1 epitopes of culturable *L. pneumophila* are shed to the environment in special phases of the life cycle [22]. To close this gap in knowledge we analysed the impact of starvation on the reactivity of different mAbs targeting outer membrane structures and thus, possible changes on the corresponding epitopes which would in turn influence the potential of VBNC *L. pneumophila* to infect host cells [6]. Moreover, the results are compared to information on culturability, viability and infectivity of the cells from parallel investigations [18, 21]. The outcome of the study is of high importance for the development of mAb-based detection techniques for culture-independent enumeration of *Legionella* in man-made water systems. Moreover, the results provide an indication for the potential significance of the outer membrane antigens as virulence factors and endotoxins of VBNC *Legionella* cells.

## Methods

### L. pneumophila strains

Four of the five tested *L. pneumophila* strains belong to SG1, three being mAb-3/1-positive according to the Dresden panel of monoclonal antibodies [8]: one clinical *L. pneumophila* strain belonging to mAb subtype Benidorm (LpClin), an environmental *L. pneumophila* strain mAb subtype France/Allentown (LpEnv), both strains are donations from A. Indra (AGES, Vienna, Austria) and one *L. pneumophila* strain mAb subtype Philadelphia (LpParis, donated by Y. Héchard, University of Poitiers, France). Additionally, we used the *L. pneumophila* strain mAb subtype OLDA (LpOlda), a SG1 but mAb-3/1-negative strain (strain collection NCTC 12008, Public Health England) and a *L. pneumophila* SG6 strain (LpSG6) (strain collection NCTC 11287). The strains were kept at − 80 °C in cryotubes (Roti-Store, Carl Roth, Karlsruhe, Germany).

### Selected monoclonal antibodies (mAbs)

MAbs from the Dresden panel, which are all raised against the LPS of *L. pneumophila*, were chosen as they are extensively tested and characterized [7, 22, 23] and were routinely used in typing assays of patient and environmental samples [8]. Among them, we used the mAbs 3/1, 8/4, 8/5, 20/1, 26/1 and 88/3, which show high reactivity to different monoclonal subtypes of culturable *L. pneumophila* SG1 strains. The mAb 82/2 was shown to bind to shed membrane vesicles of *Legionella* cells (unpublished data). MAb 32/3 reacts with the LPS of SG 2–14 strains but not with SG1, and mAb 9/1 is specific for SG6. Additionally, mAbs specific for other outer membrane structures such as MOMP (major outer membrane protein, MonoFluo™ *L. pneumophila* IFA test kit (Biorad, Vienna, Austria)), the Macrophage infectivity potentiator protein (Mip) (mAb 18/1, TU Dresden), the flagellum (mAb 48/5, TU Dresden) and for an unknown 50 kDa protein (mAb 20/3, TU Dresden) were selected for the ELISA pre-screening against culturable and nonculturable *L. pneumophila* cells (Table 1).

**Table 1:**
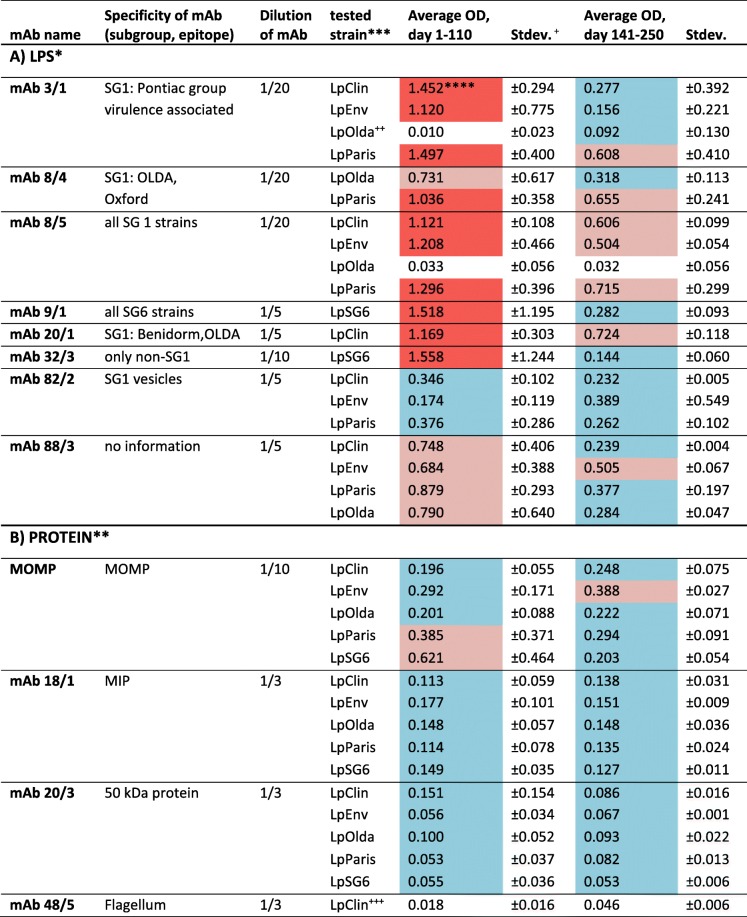
ELISA results; target strains, mAbs used and OD-values are shown as average of two periods

*values were calculated from duplicate analyses (A)

**values were calculated from two duplicate analyses (B)

***Only mAb-strain combinations resulting in positive OD-signals or for which a positive signal was expected were analysed in detail and presented here

****heat-map characteristics: red: OD > 1, strong positive reactivity; light red LPS mAbs: 0.5 < OD> 1, light red protein mAbs: OD > 0.3, positive reactivity; blue LPS mAbs: 0.05<OD> 0.5, blue protein mAbs: 0.05<OD> 0.3 weak reactivity; no colour: no reactivity

^+^Stdev.: standard deviation

^++^According to Dresden panel of mAbs LpOLDA is negative in mAb 3/1 ELISA

^+++^Results of only one strain are shown, because all strains were negative in ELISA

### Preparation of starvation microcosms

All microcosms used in ELISA and FCM were prepared in a standardized procedure described in detail elsewhere [18]. In brief, *Legionella* strains were grown in liquid buffered yeast extract (liBYE, modified [24]) and sub-cultured once, until the population reached the stationary phase, assessed by optical density (OD) measurement (OD-values of stationary phase: 3–4 depending on the strain, OD analysis was conducted after eight-fold dilution in liBYE). Then bacterial cultures were washed twice by centrifugation at 3500×*g* at 20 °C with ultrapure water (autoclaved water for laboratory use, grade 1, ISO 3696:1987 [25], Simplicity® Ultrapure water (Merck, Darmstadt, Germany)). Approximately 10^8^ cells mL^− 1^ (final concentration, using a conversion factor comparing OD values with total cell counts determined by SYBR green 1/propidium iodide staining [26] and epifluorescence microscopy (EFM) [27]) were inoculated to sterile glass bottles prefilled with 350 mL of autoclaved ultrapure water (starvation microcosms). After an initial sampling, all microcosm bottles were incubated at 45 °C without shaking and periodically sampled by pouring out 15 mL of each bottle and aliquoting the sample for further analysis [18, 21]. 500 μl were used for immunofluorescence (IF)-FCM.

### Experimental design

In order to study the persistence of outer membrane structures during short and long-term starvation, five *L. pneumophila* strains were starved in ultrapure water in batch-culture microcosms at 45 °C. First, by using an indirect enzyme-linked immunosorbent assay (ELISA) the reactivity of various mAbs to culturable and nonculturable *L. pneumophila* cells in starvation microcosms was screened after different incubation periods up to 250 days to preselect the mAbs for the IF-FCM analyses. ELISA results obtained were only exploratory and not intended to draw experimental conclusions from them. Second, for the ideal mAb-candidates IF-assays were implemented for FCM to quantitatively analyse staining patterns at the single cell level. With ELISA and IF-FCM, we only analysed mAb-strain combinations which clearly resulted in positive signals or for which a positive signal was expected (Tables 1 and 2). For the ELISA, one microcosm for each timepoint and for each strain was prepared and analysed (5–8 microcosms within 250 days per strain); for IF-FCM analyses, triplicate microcosms were prepared for each of the five *L. pneumophila* strains and analysed at 9 to 10 time points. Samples were taken once per week until complete loss of culturability (defined as < 1 CFU/mL, after approx. 8 log_10_ units decrease) plus three extra weeks (short-term starvation period). After this period, samples were analysed by IF-FCM at two to four additional time-points for up to 1 year (long-term starvation period). In parallel investigations, all samples were tested for culturability on BCYE plates (bioMérieux, Vienna, Austria), for viability and infectivity, as described elsewhere [18, 21].

**Table 2:** Assay conditions and strains for the different mAbs used for IF-FCM analysis

Antibody	Tested strains^a^	antibody class	Final dilution	Incubation temperature	Incubation time
MAb 3/1	LpParis, LpClin, LpEnv	IgG	1/20	37 °C	90 min
MAb 8/4	LpParis, LpOlda	IgG	1/20	37 °C	90 min
MAb 8/5	LpParis, LpClin, LpEnv, LpOlda	IgM	1/20	37 °C	90 min
MAb 9/1	LpSG6	IgM	1/5	37 °C	90 min
MAb 20/1	LpClin	IgG	1/5	4 °C	60 min
MAb 26/1	LpOlda	IgG	1/10	37 °C	90 min
MAb 32/3	LpSG6	IgG	1/10	37 °C	90 min
Monofluo kit	All strains	–	1/10	37 °C	60 min
Second. anti-IgG or IgM-FITC Ab		–	1/90	37 °C	30 min

^a^Only those strain-mAb combinations were investigated which showed positive signals in ELISA

### Indirect ELISA

For the analysis of LPS-targeting mAbs, all *Legionella* samples were heat-inactivated at 95 °C for 10 min; the other ELISAs were conducted with living *Legionella* cells to preserve target surface structures. The protocol for indirect ELISA [7] was slightly modified. 96-well polystyrene plates (Greiner Bio-One, Frickenhausen, Germany) were coated with 50 μL of starvation microcosm samples per well and incubated for 2 h at 37 °C or overnight at 4 °C in a humidified dark chamber. For each sample/LPS-mAb combination two wells were coated: one each on duplicate polystyrene plates; for each sample/protein-mAb combination four wells were coated: two each on duplicate polystyrene plates. After washing three times with 400 μL PBS per well, 200 μL blocking buffer (PBS + 10% fetal calf serum (FCS)) was applied for 1 h at 37 °C to block unspecific binding sites. After a second washing step, 50 μL of the primary mAb (provided as culture-supernatant) diluted in blocking buffer (dilutions see Table 1) was added and incubated for further 1.5 h. The plates were washed again three times and 50 μL of the 1/200 diluted goat anti-mouse HRP-conjugated antibody (IgG + IgM, reacting with heavy and light chains (H + L), Pierce Biotechnology, Rockford, USA) was added to each well and incubated for 1 h at 37 °C. After a further washing step, 50 μL of substrate solution (3,3′,5,5′-tetramethylbenzidine Liquid Substrate, slow kinetic form, Sigma Aldrich, St.Louis, USA) was applied to the wells at room temperature in the dark for 10 and 15 min to analyse LPS mAbs and mAbs targeting other outer membrane components, respectively. The reaction was stopped by adding 50 μL 1 M hydrochloric acid. The extinction of the colour-reaction was measured photometrically at 450 nm wavelength and at 620 nm as reference wavelength to correct for background due to autofluorescence/luminescence or spill-over into other channels. One line of the 96-well-plate was always dedicated to the negative control and coated with PBS as antigen only. From all delta OD 450/620 values the respective negative control values were subtracted. One replicate microcosm was analysed for each point of time and strain, all samples were analysed in the same ELISA experiment to minimize inter-ELISA variability (Table 1).

### Immunofluorescence analyses with flow cytometry

IF-FCM subsamples from each triplicate microcosm were stained with the LPS-mAbs tested positive in the ELISA and with the MOMP-mAb (Table 1). To implement the method, different incubation conditions for each mAb-strain combination were tested by FCM and visually by epifluorescence microscopy. In the resulting standardised protocol, microcosm subsamples, mAbs from the Dresden panel (culture supernatants) and the secondary FITC-labelled antibody-conjugates IgG or IgM (goat anti-mouse IgG (H + L)-FITC-conjugate or goat anti-mouse IgM-FITC-conjugate, Pierce Biotechnology) were diluted in autoclaved, filter sterilized PBS (prepared in ultrapure water) supplemented with 1% bovine serum albumin (30% BSA, PAA Laboratories Inc., Pasching, Austria) (PBS-BSA). All antibodies used, dilutions, staining conditions and strain-antibody combinations are detailed in Table 2. Microcosm samples were diluted 1/2 (100 μL sample + 100 μL PBS-BSA). The same volumes of mAbs and samples (20 μL each) were mixed and incubated at either 37 °C or 4 °C for 90 or 60 min, respectively. Afterwards, the same volume of the diluted secondary FITC-labelled mAb was added and incubated for further 30 min at 37 °C or 4 °C without washing steps. In the MOMP staining procedure the diluted subsamples were mixed directly with 2 μL of the undiluted MOMP staining reagent of the MonoFluo™ kit and incubated for 1 hour at 37 °C. Finally, for all analyses, the sample was filled up to 1 mL volume with cold, sterile filtered PBS to obtain a final cell concentration of approximately 1 × 10^6^ cells mL^− 1^ (final dilution 1:100) and analysed immediately by FCM or stored until FCM analysis at 4 °C for not more than 2 hours. For each strain and antibody, three controls were added for the FCM analyses: (i) a background control consisting of stained PBS-BSA only, (ii) a stained negative control using a *Legionella* strain which was considered negative for the respective mAb and (iii) a sample stained with the secondary antibody FITC-conjugate only to control the unspecific staining of the secondary antibody.

#### Flow cytometry analysis

An Attune Nxt flow cytometer (Life Technologies, Darmstadt, Germany) equipped with a 488 nm flat-top laser at 50 mW was used to perform the IF analyses. Photomultiplier tube voltages were adjusted to 700 V for forward scatter (FSC), 500 V for side scatter (SSC), 360 V for fluorescence channel 1 (FL1, 530 ± 15 nm) and 540 V for fluorescence channel 3 (FL3, 695 ± 20 nm). The threshold was set for FSC to 100 and for SSC to 1100. Samples were measured at a velocity of 100 μL min^− 1^ (2000–5000 counts/s) and for each sample aliquot 100 μL were analysed three times in a row without removing the sample from the injection port. Cell concentrations were automatically calculated by the Attune Nxt analysis software (v2.4, Life Technologies). A sequential gating procedure was applied. First, the whole cell population was gated in the FSC-H (height) versus SSC-H plot as “cells”-population (Additional file 1: Figure S2). As aggregates appeared like in the study of Wallner et al. [28], the gating strategy was adapted to fit microscopic counts. For that purpose, aggregates, cell numbers of the aggregates and single cells of stained and filtered samples (Isopore membrane filters, 0.2 μm, Merck Millipore Ltd., Tullagreen, Ireland) were counted in EFM and results were compared to the FCM results of different gating strategies concerning aggregate discrimination. The gating strategy fitting best to the microscopic results was applied: in a FL1-H versus FL1-A (area) plot of the “cells” population. “Aggregate”- and “single”- cell events were discriminated and further analysed in separate FL1-H histograms by gating positively stained cells against the unspecific staining control (example plots see Additional file 1: Figure S2). In the final calculations, the positively stained single cell concentration plus two times the positively stained aggregate concentration (average cells/aggregate in EFM = 2.3) were summed up to the final cell concentration. In most strain-antibody combinations in the FCM histograms, two peaks of positive stained cells were observed with different mean fluorescence intensities (MFI). The population of the right peak characterised by brighter staining was called “strongly-stained cells”, the left positive peak “weakly-stained cells” and both peaks taken together were termed “stained cells” (Additional file 1: Figure S2 c-f). The calculated limit of quantification was 1.3 × 10^4^ cells mL^− 1^ for all FCM analyses.

### Viability indicators and total cell count

Different viability indicators, the total cell count and the culturability of the starved legionellae were analysed in parallel investigations [18] in sample aliquots of the same triplicate microcosms as used for IF-FCM analysis. A detailed description of the used materials and methods and of the results is published in Schrammel et al. [18] and the results of each viability indicator are displayed in (Additional file 1: Figure S3) for all *L. pneumophila* strains. In principle, to indicate viability, 5,6-carboxy-fluorescein-diacetate (CFDA) staining and SG1/PI staining was applied. CFDA stained cells were interpreted as esterase- (metabolically-) active cells with an intact cell membrane. SG1/PI stained cells showed different fluorescing colours depending on their membrane integrity: green cells were interpreted as cells with intact cell membranes, orange cells as cells with intermediate membranes and red cells as cells with permeabilised membranes. The total cell count was measured with FCM after staining with SG1.

### Statistics

Correlations between the immunofluorescence and the viability data (data of each triplicate microcosm of all strains) were calculated. As most of the data did not follow a normal distribution, the non-parametric Spearman-test for correlation was applied (SPSS vers.24, IBM internat.). After Bonferroni-correction, *p*-values lower than 0.05 were accepted as significant results.

## Results

**Fig. 1:**
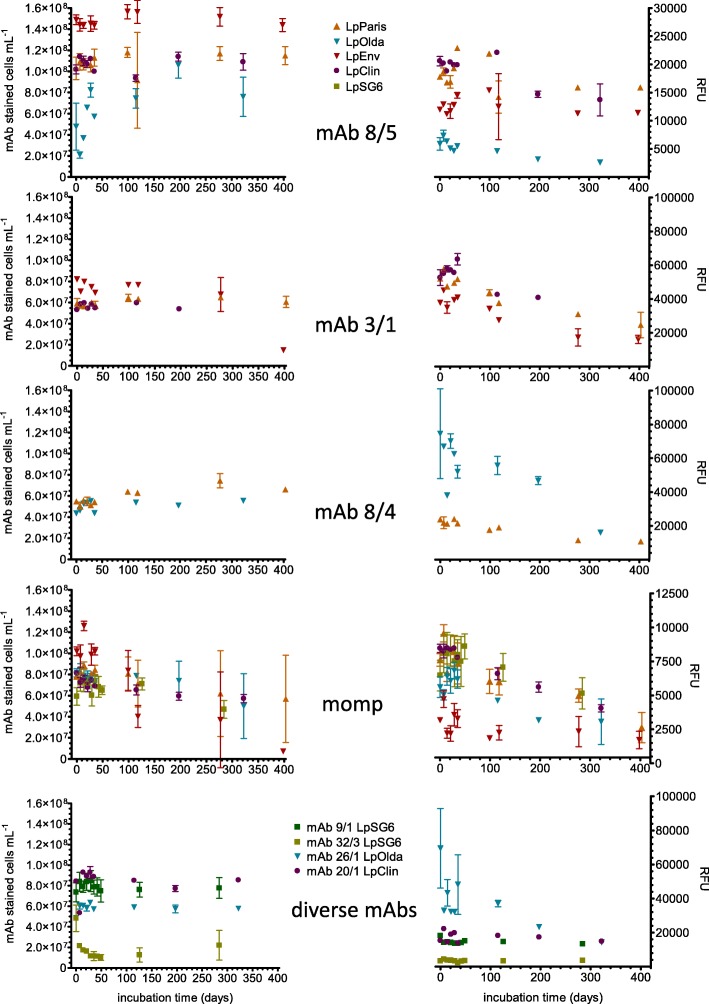
Concentration of mAb-stained cells of all tested strains (left panel) and mean fluorescence intensity (MFI) of mAb stained cells of all strains (right panel) displayed as relative fluorescence units (RFU) during 400 days of starvation. Error bars show standard deviations of triplicate microcosms. For all *L. pneumophila* serogroup 1 strains culturability was only observed at the first two time-points (day 0 and day 7), the serogroup 6 strain was culturable up to 24 days

Loss of culturability to levels below 1 CFU mL^− 1^ (8 log_10_ reduction) was already observed after 11 days for LpEnv, after 14 days for LpParis, LpClin and LpOlda and after 29 days of starvation in ultrapure water for LpSG6 (Additional file 1: Figure S1, data taken from [18]). By ELISA and IF-FCM, all analysed epitopes were detectable in the culturable and in the nonculturable state during the whole starvation period (Fig. 1). Depending on the strain-antibody combination, variations in the concentration of mAb stained cells, determined by IF-FCM, ranged from 93% decrease (LpEnv-MOMP) to 60% increase (LpOlda-mAb 8/5) compared to the initial values of 1.0 × 10^8^ and 4.8 × 10^7^ cells mL^− 1^, respectively (Table 3). Throughout the period of starvation, the total number of cells remained broadly constant ranging between 6.4 × 10^7^ cells/mL and 1.1 × 10^8^ cells/mL, with standard deviations of 9.6 × 10^6^ to 1.4 × 10^7^ depending on the strain [18].

**Table 3:**
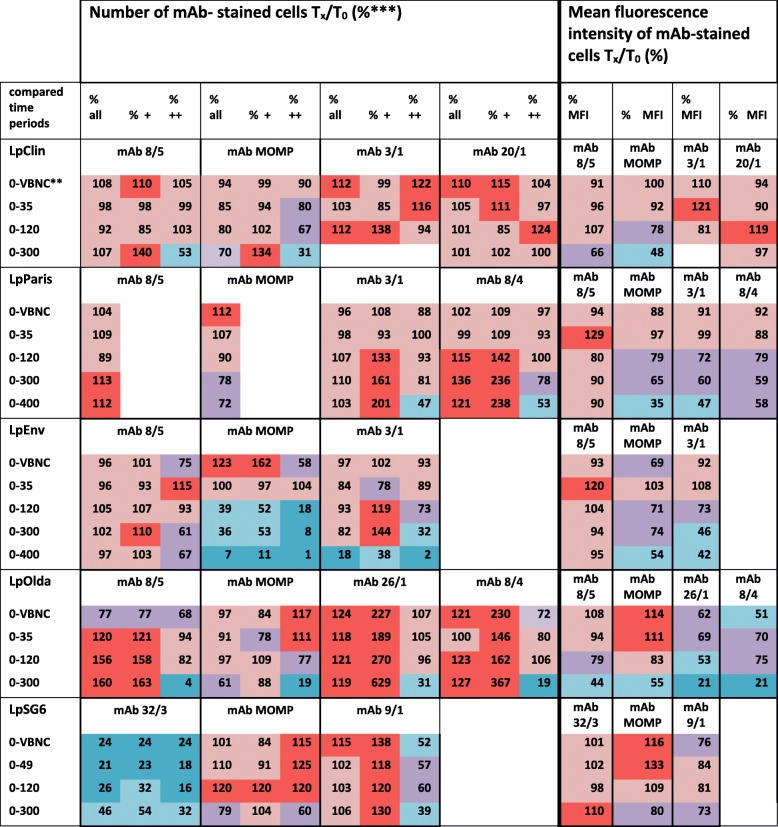
Heat-map* of percentage of number and of MFI of mAb-stained-cells in relation to day 0

*Heat-map colors: Dark blue: 1–30%; light blue: 31–55%; violet: 56–85%. light red: 85–110%; red: ≥ 110%;

**Each row shows a certain time-period, which is given in approximate day-periods. 0-VBNC: period of day 0 until complete loss of culturability (< 1 CFU/mL)

***Percentage is differentiated in (i) all stained cells (all), (ii) weakly (+) and (iii) strongly (++) stained cells

### ELISA results

Results of ELISA and categorisation rules are described in Table 1. In general, for all LPS mAbs we observed on average higher OD-values in the first 110 days of starvation in comparison to the later time-points from 141 to 250 days. The differences between the two periods were not statistically significant, as standard-deviations of the mean OD-values were high (Table 1A). According to the Dresden panel of mAbs, LpOlda should react strongly with mAb 8/5, however our results showed that this mAb bound to LpOlda only at rare time-points at a weak OD below 0.2. The mAb 82/2, which was found to bind to shed vesicles in previous experiments (unpublished data) exhibited constant weak reactivity to the epitopes of all strains throughout the whole starvation experiment. All ELISA results of the protein-mAbs were lower compared to LPS-mAb results, but positive at all tested time-points (Table 1B).

### Immunofluorescence-FCM results

#### Concentration of mAb-stained cells

For most of the antibody-strain combinations, concentrations of mAb-stained cells analysed with FCM were stable from the day zero of inoculation until 200–300 days of starvation and even longer, with only small fluctuations (Table 3, Fig. 1). The epitopes of the two isotype-IgM antibodies mAb 8/5 (specific for all *L. pneumophila* SG1 strains) and mAb 9/1 (specific for LpSG6) were constantly present without major changes in cell numbers over the whole starvation experiment for three out of four LpSG1 strains and for the LpSG6 strain, respectively. One exception was observed: mAb 8/5-positive-stained LpOlda cells increased by a factor of two from day 0 until day 197. The same trend of persistent IF-staining throughout the experiment was found for the mAb 20/1-stained cells of LpClin, the mAb 26/1-stained cells of LpOlda and the mAb 3/1-stained populations of all tested strains (LpParis, LpEnv, LpClin) carrying the virulence-associated epitope. At day 398, only the mAb 3/1-stained cells of LpEnv decreased drastically to 18% of the initial value (8.2 × 10^7^ cells mL^− 1^).

The mAb 8/4 exhibited some different patterns with an increase in the beginning of the experiment. The number of stained LpOlda cells increased in the first 2 weeks by approximately 20% of the initial value (4.4 × 10^7^ cells mL^− 1^) whereas the number of stained LpParis cells increased by ~ 30% of the initial value (5.5 × 10^7^ cells mL^− 1^) until 99 days of starvation. On the contrary, the LpSG6 cells stained with mAb 32/3 decreased along with culturability. Highest numbers were observed at day 0; 1 week later, the mAb 32/3 stained cell number already declined to 44% of the initial concentration (4.8 × 10^7^ cells mL^− 1^) and stabilized at day 29 after the complete loss of culturability at around 24%.

A constant number of MOMP-stained cells was found for all strains before 300 days of starvation; later, the cell numbers declined to 60–80% of the initial values (LpParis 7.9 × 10^7^ cells mL^− 1^, LpClin 8.1 × 10^7^ cells mL^− 1^, LpOlda 8.2 × 10^7^ cells mL^− 1^ and LpSG6 5.9 × 10^7^ cells mL^− 1^) except for LpEnv (Table 3). MOMP-stained LpEnv cells decreased already at day 118 to 50%, and thereafter continuously to 7% of the initial concentration (1.0 × 10^8^ cells mL^− 1^) after 400 days.

#### Epitope development - mean fluorescence intensity (MFI)

The MFI can be considered as equivalent to the average amount of epitopes on the cell surface available for antibody binding. Overall, the MFI was less stable than the concentration of stained cells and was highly dependent on the antibody-strain combination (Table 3, Fig. 1). The highest decrease in the MFI was observed for the mAb 26/1-stained LpOlda cells. Already during the first week of starvation, it dropped to 50–70% of the initial value (70,000 relative fluorescence units, RFU) and further decreased to 20–30% until the end of the experiment, whereas the MFI of mAb 8/5-stained LpOlda showed a continuous decrease. The MFI of the mAb 8/5-stained LpEnv and LpParis cells was very stable throughout the experiment, so was the MFI of the mAb 20/1-stained LpClin cells and the MFI of LpSG6 cells stained with all mAbs (Table 3, Fig. 1).

The MFI of the MOMP and mAb 3/1-stained cells (both epitopes are virulence related) were significantly inter-correlated (rho = 0.868, *p* < 0.05). Already after the short-term period they decreased to around 70–80% of the initial value (MOMP MFI: LpEnv 3200 RFU, LpParis 7600 RFU, LpClin 8500 RFU, LpOlda 5600 RFU; mAb 3/1 MFI: LpEnv 37,800 RFU, LpParis 52,000 RFU, LpClin 52,500 RFU) and further declined until 55% after 300 days of starvation, except for LpSG6. The MFI of MOMP stained LpSG6 cells was constant until 200 days of starvation and decreased slightly at the last time-point to 80% of the initial value (6500 RFU) (Table 3, Fig. 1).

#### Strongly- and weakly-stained cells

Three different patterns were observed regarding the fluorescence intensity of the stained cells during starvation (as an example see FCM plots in Additional file 1: Figure S2). First, the number of strongly-stained cells with a lot of binding sites decreased throughout the experiment, while the number of weakly-stained cells increased and exceeded the number of strongly-stained cells in the long-term period between 100 and 200 days of starvation. The mAbs MOMP (except for LpOlda), 3/1, 8/4 and 26/1 exhibited this pattern (Table 3). Second, the number of weakly-stained cells was constant throughout the experiment and the same or higher than the strongly-stained cells. This was true for mAb 8/5, 32/3, 9/1, 20/1 and MOMP (only for LpOlda) (Table 3). Third, for the mAbs strain combination MOMP/LpParis and mAb 8/5/LpParis no strongly-stained cells were observed.

#### Presence of epitopes compared to viability

The ratio between viable cell counts and mAb-stained cell counts was calculated for two viability indicators (data taken from [18], displayed in Additional file 1**:** Figure S3 A-D): (i) the number of esterase active cells and (ii) the sum of cells with intact and with intermediate membranes (Table 4). Generally, a high percentage of all mAb stained cells was viable for all strains, at least until day 118. In most cases, the percentage was smaller than 100% (Table 4, blue fields), indicating that not all of the mAb-stained cells showed signs of viability. This is especially true for the mAb 3/1-positive strains and LpSG6, after all cells became unculturable. Interestingly, at time-point zero, the number of viable cells of LpParis, LpOlda and LpSG6 was higher than the number of most mAb stained cells. For most strains, the percentage of mAb-stained viable cells decreased with increasing starvation time. At the latest time-point, viable cells partly declined to less than 1% of the epitope carrying population. LpOlda displayed a completely different picture: higher viable cell numbers than mAb-stained cell numbers (> 100%) concerning the LPS epitopes for up to 200 days of starvation, especially when membrane integrity was used as viability indicator. Percentages > 100% were also observed for mAb 32/3 with LpSG6 at all time-points for both viability indicators and for mAb 3/1 with LpEnv during short-term starvation when membrane integrity was used as viability indicator.

**Table 4:**
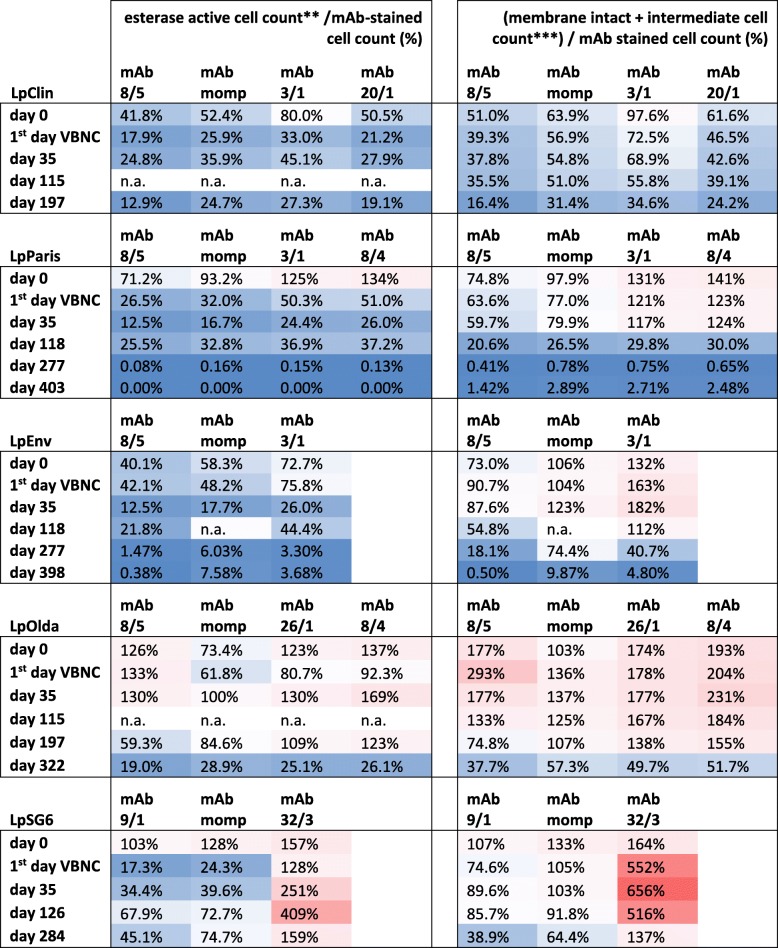
Heat-map* of the ratio between viable cells (data taken from [18]) and mAb-stained cells

*percentages > 100% are displayed in red and percentages < 100% in blue; Percentages higher than 100% indicate that only a part of the viable cells were expressing the epitope recognized by the antibody tested

**esterase active cells were determined by CFDA-staining and FCM analysis [18]

***membrane intact and membrane-intermediate cells were determined by SYBR green1/Propidium iodide staining and FCM analysis [18]

## Discussion

In man-made water systems legionellae are usually detected by standard culture-based methods. These methods may overlook potentially dangerous *Legionella* cells in these systems, when they encounter stress conditions and enter into the VBNC state. To analyse the impact of stress imposed by starvation on the outer membrane surface structures relevant as diagnostic recognition sites and as major immuno-dominant antigens, we analysed the mAb-reactivity patterns of five *L. pneumophila* strains incubated for 1 year in ultrapure water at 45 °C.

### Despite loss of culturability, outer membrane epitopes are persistently present during short- and long-term starvation

The ELISA was obviously a good method of choice for pre-screening the reactivity of the mAbs against starved *Legionella* cells of different age. IF-FCM analyses allowed the quantification of surface epitopes at the single cell level. A diversification of the *Legionella* populations during starvation was found due to changes in the outer membrane structure of some of the epitopes (Fig. 1**,** Tables 3 and 4). Generally, the IF-FCM analyses of mAb 3/1, 8/5, 9/1, 20/1, 26/1 and MOMP showed mainly stable cell concentrations during the first 100 to 200 days, followed by a slight decrease for some mAb-strain combinations. These results are in line with the results from another study, which examined the LPS stability of VBNC *Aeromonas salmonicida* bacteria during conditions of starvation. The authors observed no major changes in the LPS profiles of the starved VBNC cells compared to culturable ones [29]. In our study, the number of cells stained by the mAbs 3/1, 8/5, 9/1, 20/1, 26/1 and MOMP were not related to the decreasing number of viable cells analysed in the parallel investigations [18] (Table 4 and Additional file 1: Figure S3). Obviously, not only viable cells were stained by the mAbs, but the epitopes were also present on the surface of dying cells. However, generally a large percentage of the mAb stained cells was viable at least for approximately 115 days of starvation. In this context, the sum of intact and intermediate membrane cells better reflected the number of mAb stained cells than the esterase active cells, as the first two parameters are connected to the functionality of the cell membrane. In some mAb-strain combinations the number of viable cells was larger than the number of mAb stained cells (Table 4, values > 100% and Additional file 1**:** Figure S3). Especially the LpOlda strain was stained with a low efficiency for all LPS-mAbs until 118 days of starvation. We can only speculate about the reasons. Most probably, this strain has degenerated LPS structures. With the ELISA we found low binding affinities already in the culturable state. Generally, percentages higher than 100% indicate that viable cells were not expressing the epitope recognized by the antibody tested. This could be interpreted as an active re-modelling of the cell envelope, or a degradation of the molecule carrying the epitope, or a combination of both.

In contrast to the long-term stability of the mAb-stained populations described above, for specific mAbs obvious changes over time were observed. However, all these changes were in the range of maximum 1 log_10_ unit of the initial cell concentration for the whole experimental period (with the exception of MOMP/LpEnv with 93% reduction), while the number of culturable cells decreased by 8 log_10_ units and the number of membrane-intact, metabolically active cells by up to 4 log_10_ units after ~300 days of starvation. One study investigated the reactivity of a polyclonal antibody against membrane epitopes of *Vibrio parahaemolyticus* in the transition to VBNC state triggered by cold temperature, and observed a 1 log_10_-reduction in antibody-stained cell numbers at day 29 of incubation followed by stable counts of stained cells during the transition phase to the VBNC state [30]. Another study tested an electrode coated with a mAb (no information given about target-epitope specification) for the detection of culturable and heat-induced VBNC *L. pneumophila* and obtained higher detection results for VBNC than culturable cells [31]. From these studies and our results it can be concluded that the physiological state of the cells impacts the LPS only at specific sites, such as the mAb 32/3 and 8/4 epitopes. Other outer membrane epitopes of *L. pneumophila* are persisting in a constant population throughout the culturable and the nonculturable state during a starvation period for up to 1 year. However, we could show, that at the same time the number of mAb-target binding sites per stained cell for mAb 3/1, 8/4, 26/1 and MOMP decreases (decreasing MFI). We hypothesize that the stably-expressed epitopes like the mAb 8/5, 9/1, 20/1 and 26/1 belong to the more basic structure of the LPS outer core or O-antigen, whereas the other epitopes, which get modified during starvation, represent components that are more distal from the core region or smaller alterations of the LPS and get degraded or modified during long-term starvation.

### The link between bacterial cell viability and MFI was stronger than the link between viability and number of mAb-stained cells

Significant negative correlations between specific mAb stained cell concentrations and the viable cell concentrations were observed (Additional file 1: Table S1). In the course of starvation, the accessibility of the mAb 8/4 epitope may have improved due to conformational changes or due to the loss of LPS side-chains while membrane-integrity and highly esterase-active cells concomitantly decreased (rho = -0.579 and -0.519, respectively, *p* < 0.001). In contrast, the mAb 32/3-stained cell concentration of LpSG6 significantly correlated positively with the number of highly esterase-active cells (rho = 0.759, *p* < 0.001) (Additional file 1: Table S1). We hypothesize that the epitope for this mAb is only expressed in highly active cells. More significant correlations of the viability data were found with the MFI of the different mAb-strain combinations than with the number of mAb-stained cells (Additional file 1: Table S1). From this data it can again be concluded, that these outer membrane epitopes are reduced or rebuilt when viability (analysed in terms of membrane integrity and high esterase activity) declines. All the other epitopes (mAb 8/5, 9/1, 26/1 and 20/1) are obviously not bothered by changes in viability or not to a significant extent during long-term starvation.

### The presence of outer membrane epitopes alone is not a sign for infectivity

The LPS of *L. pneumophila* is known to be the immuno-dominant antigen and for its endotoxic activity in Legionnaire’s disease [4, 5]. Legionellae possessing the mAb 3/1 epitope are disproportionally more often related to clinical cases than mAb 3/1 negative strains [8]. The MOMP was mentioned to be an important factor for the phagocytosis of human macrophages [10]. During starvation, these virulence related factors show long-term stability, whereas the infectivity towards eukaryotic host cells (co-cultures of starved legionellae with amoebae and human macrophages conducted in the parallel investigation [21]) decreased already markedly with the loss of culturability during short-term starvation. Thus, we conclude, that not all *Legionella* cells of a starved population, which possess the virulence-related MOMP and mAb 3/1 epitopes might be infective. The presence of those epitopes alone is not a direct indication of virulence, but might be a prerequisite.

## Conclusions

There is a clear need in water surveillance for the standardisation of culture-independent direct methods for the detection of both culturable and VBNC *Legionella* cells in environmental samples [17]. MAb-based techniques such as IF-FCM or IF-solid-phase cytometry in combination with viability staining [12, 16] have been proposed for the specific and culture-independent enumeration. Furthermore, immuno-magnetic separation systems [12, 13] (e.g. from rqmicro) for the isolation of environmental *L. pneumophila* cells, particularly from samples with high microbial background, were recently introduced**.** The results of this study demonstrate the general applicability of antibody-based techniques for the detection of starved VBNC legionellae. Most of the mAbs tested stained culturable cells and unculturable cells in a comparable way with a high percentage of viable (VBNC) cells. IF-FCM proved to be a valuable method for the single-cell analysis of reactivity patterns of mAbs against *L. pneumophila* cells, as the MFI of the stained cells was at least one log_10_ unit above the MFI of the negative control. Additionally, we tested two of these mAbs for immuno-magnetic separation (IMS) of spiked *Legionella *VBNC cells and found recovery rates around 95% [32] supporting the results of other studies [12, 13]. With this study we show, that most of the tested LPS epitopes (mAb 3/1, 8/5, 9/1, 20/1, 26/1) and the MOMP epitope are persistently present in long-term starved *L. pneumophila* cells. However, the presence of these epitopes is not necessarily related to the viability and the infectivity of the cells. Thus, mAb-based detection techniques might overestimate the number of viable cells. Nevertheless, they are valuable tools when used in combination with viability indicators or other downstream analyses to reliably detect all potentially health relevant *L. pneumophila* cells in engineered water systems. Of course, such detection methods based on mAbs have to be carefully evaluated for a variety of strains and for diverse conditions to reliably detect all present and living cells, because water system disinfection strategies such as heat, the use of biocides or natural conditions such as nutrient depletion, could potentially have an adverse effect on the targeted outer membrane epitopes.

## Additional file

Additional file 1: **Figure S1.** Culturability of five *L. pneumophila* strains during starvation [18]. **Table S1**. Spearman’s rank correlation results of the IF-FCM-data and the viability data (taken from the parallel investigations [18]). **Figure S2.** Example plots for IF-FCM analysis (mAb 8/4 and mAb MOMP) of starved *Legionella* cells and gating strategy after IF staining. **Figure S3.** Viability indicator data for the five *L. pneumophila* strains examined during starvation in ultrapure water at 45 °C for up to 400 days [18]. (DOCX 868 kb)

## Abbreviations

CFDA: 5,6-carboxy-fluorescein acetate
CFU: Colony forming units
EFM: Epifluorescence microscopy
ELISA: Enzyme linked immuno-sorbent assay
FCM: Flow cytometry
IF: Immuno-fluorescence
IMS: Immuno-magnetic separation
liBYE: Liquid buffered yeast extract
Lp: *L. pneumophila*
LPS: Lipopolysaccharide
mAb: Monoclonal antibody
MFI: Mean fluorescence intensity
Mip: Macrophage infectivity potentiator
MOMP: Major outer membrane protein
OD: Optical density
RFU: Relative fluorescence units
SG: Serogroup
SG1/PI: SYBR green 1/propidium iodide
st: Short-term
stdev: Standard deviation
VBNC: Viable but nonculturable
