# Supplementary material for: Persistent presence of outer membrane epitopes during short- and long-term starvation of five Legionella pneumophila strains
Source: BMC Microbiol. 2018 Jul 17;18:75. doi: 10.1186/s12866-018-1220-x (PMC6050704; doi:10.1186/s12866-018-1220-x)
Supplement: Supplementary file 1 — Figure S1. Culturability of five L. pneumophila strains during starvation [18]. Table S1. Spearman’s rank correlation results of the IF-FCM-data and the viability data (taken from the parallel investigations [18]). Figure S2. Example plots for IF-FCM analysis (mAb 8/4 and mAb MOMP) of starved Legionella cells and gating strategy after IF staining. Figure S3. Viability indicator data for the five L. pneumophila strains examined during starvation in ultrapure water at 45 °C for up to 400 days [18]. (DOCX 868 kb) [file 12866_2018_1220_MOESM1_ESM.docx]

Schrammel B, Petzold M, Cervero-Aragó S, Sommer R, Lück C, Kirschner AKT: **Persistent presence of outer membrane epitopes during short- and long-term starvation of five *Legionella pneumophila* strains**

# Additional file

**Figure S1.** Culturability of four *L. pneumophila* SG1 strains and one SG6 strain during conditions of starvation in ultrapure water at 45°C for up to 400 days. Log_10_-transformed average values of triplicate microcosms are shown, error bars depict standard deviations. All 0-values correspond to values < 1 CFU/mL. Data taken from [1].

**Table S1.** Spearman's rank correlation of the IF-FCM results and the viability data (taken from [1]), values of rho (correlation coefficient) are shown; Bonferroni correction was applied. Only significant results are shown. Only strains which were specific and tested for the respective mAb are included for the calculation of statistics:

mAb 3/1: strains LpParis, LpEnv, LpClin

mAb 8/4: strains LpParis, LpOlda

MOMP: all tested strains

mAb 32/3: strain LpSG6

| **Rho** | **mAb stained cell concentration (FCM)** | | | **Mean fluorescence intensity (MFI) of mAb-stained cells** | | |
| --- | --- | --- | --- | --- | --- | --- |
| **Viability data taken from [1]** | mAb 8/4 | mAb MOMP | mAb 32/3 | MFI mAb 3/1 | MFI mAb 8/4 | MFI mAb MOMP |
| highly esterase-active cells^+^ | -.519** | - | .759** | .523** | .433* | - |
| intact-membrane cells ^++^ | - | - | - | .548** | .731** | .338** |
| intact plus intermediate membrane cells^+++^ | -.597** | - | - | - | .829** | - |

*… P ≤ 0.05

**… P ≤ 0.01

^+^ numbers of cells stained with CFDA exhibiting high fluorescence, interpreted as cells with high esterase (metabolic) activity.

^++^ numbers of cells stained with SG1/PI exhibiting green fluorescence, interpreted as cells with intact membrane.

^+++^ sum of number of cells stained with SG1/PI exhibiting green and orange fluorescence, interpreted as cells with intact and intermediate (partly permeabilized) cell membrane.


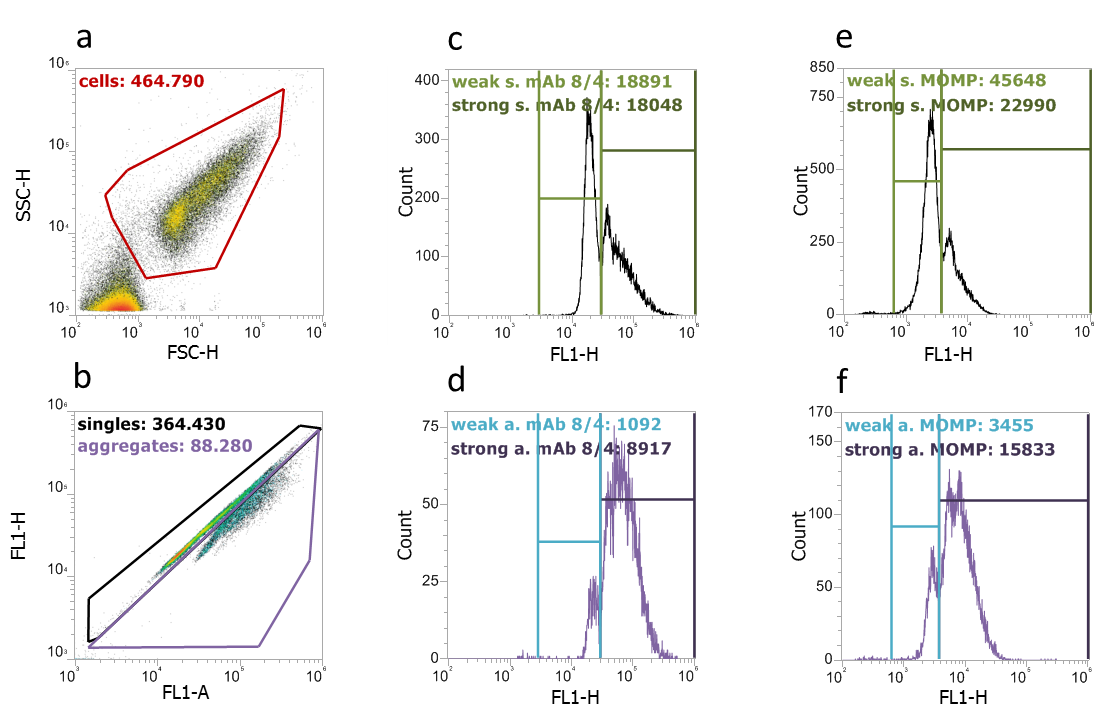


**Figure S2.** Example plots for flow cytometry analyses of starved *Legionella* cells and gating strategy after immunofluorescence staining with diverse mAbs. **a**: Gated „cell“ population in FSC versus SSC-height plot of LpParis after 14 days of starvation. **b**: Doublet discrimination in the fluorescence channel (FL) 1-height (H) versus FL1-area (A) plot of LpParis at timepoint 14 days of starvation. **c** and **d**: Histogram of mAb 8/4-stained cells of LpParis after 14 days of starvation; **e** and **f:** MOMP staining of LpEnv after 7 days of starvation; **c-f:** left gate for weakly mAb-stained cells, right gate for strongly mAb-stained cells; **c, e:** “single” (s.) - gated events; **d, f**: “aggregate” (a.) - gated events; numbers are counts in 100 µL diluted sample (1/100).

**Figure S3.** Viability indicator data for the five *L. pneumophila* strains examined during starvation in ultrapure water at 45°C for up to 400 days for the parameters **A:** low esterase active cells, **B:** highly esterase active cells (both after staining with CFDA, analysed by FCM), **C:** cells with intact membranes, **D:** cells with intermediate-damaged membranes (both after double-staining with SYBR green I and propidium iodide, analysed by FCM) and **E:** total cell count (stained with SYBR green I, analysed by FCM). Log_10_-transformed average values of triplicate microcosms are shown, error bars depict minimum and maximum values. All data are taken from elsewhere [1].

| 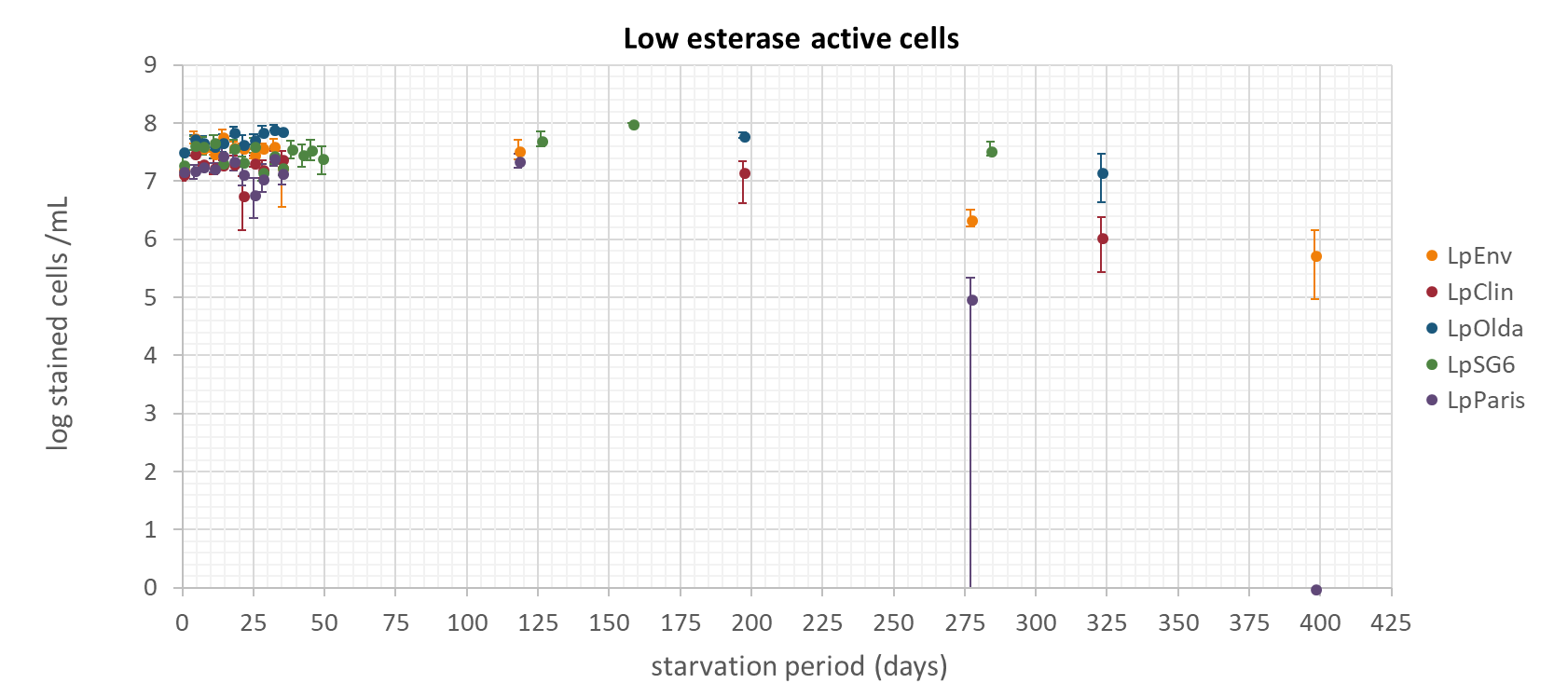  A |
| --- |
| 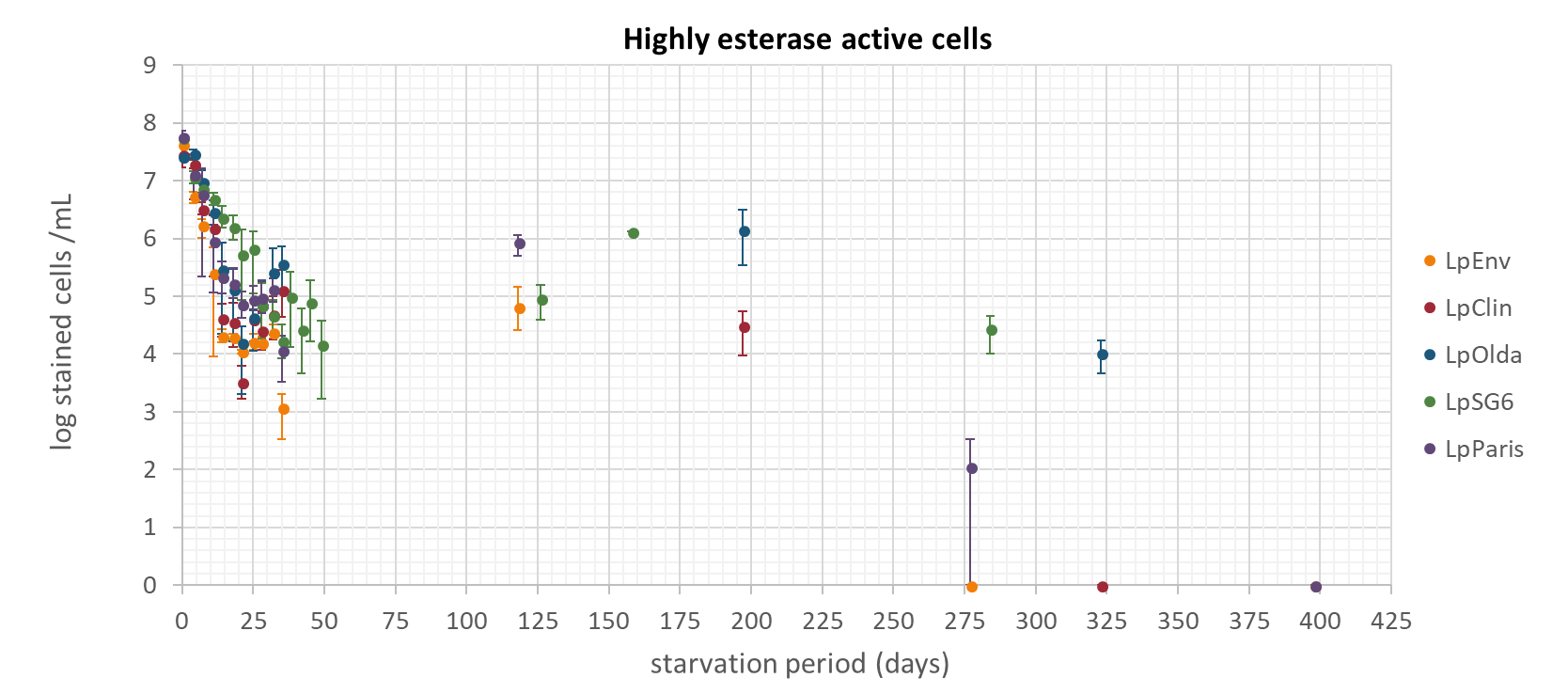  BB |
| 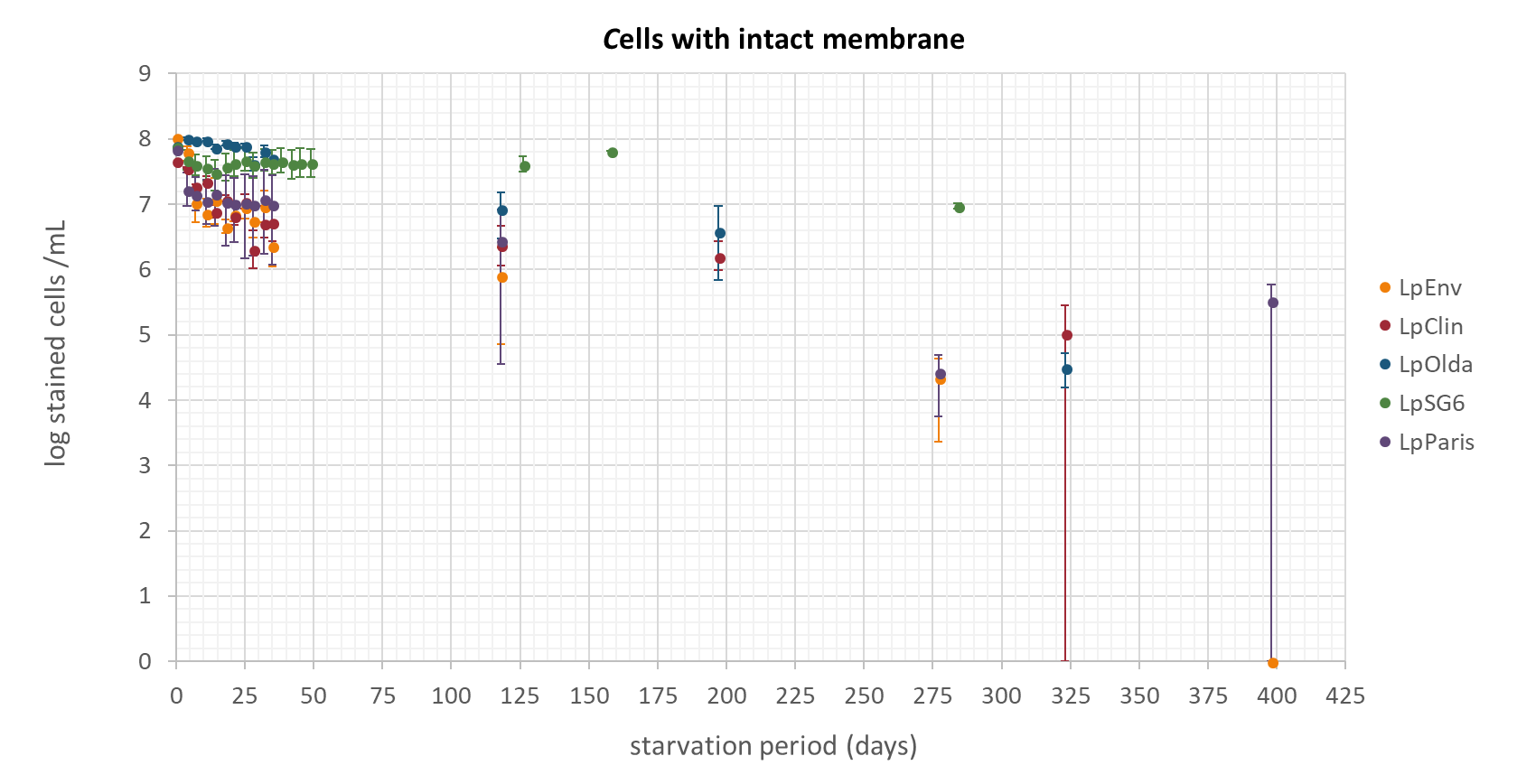  C |
| DD |
| 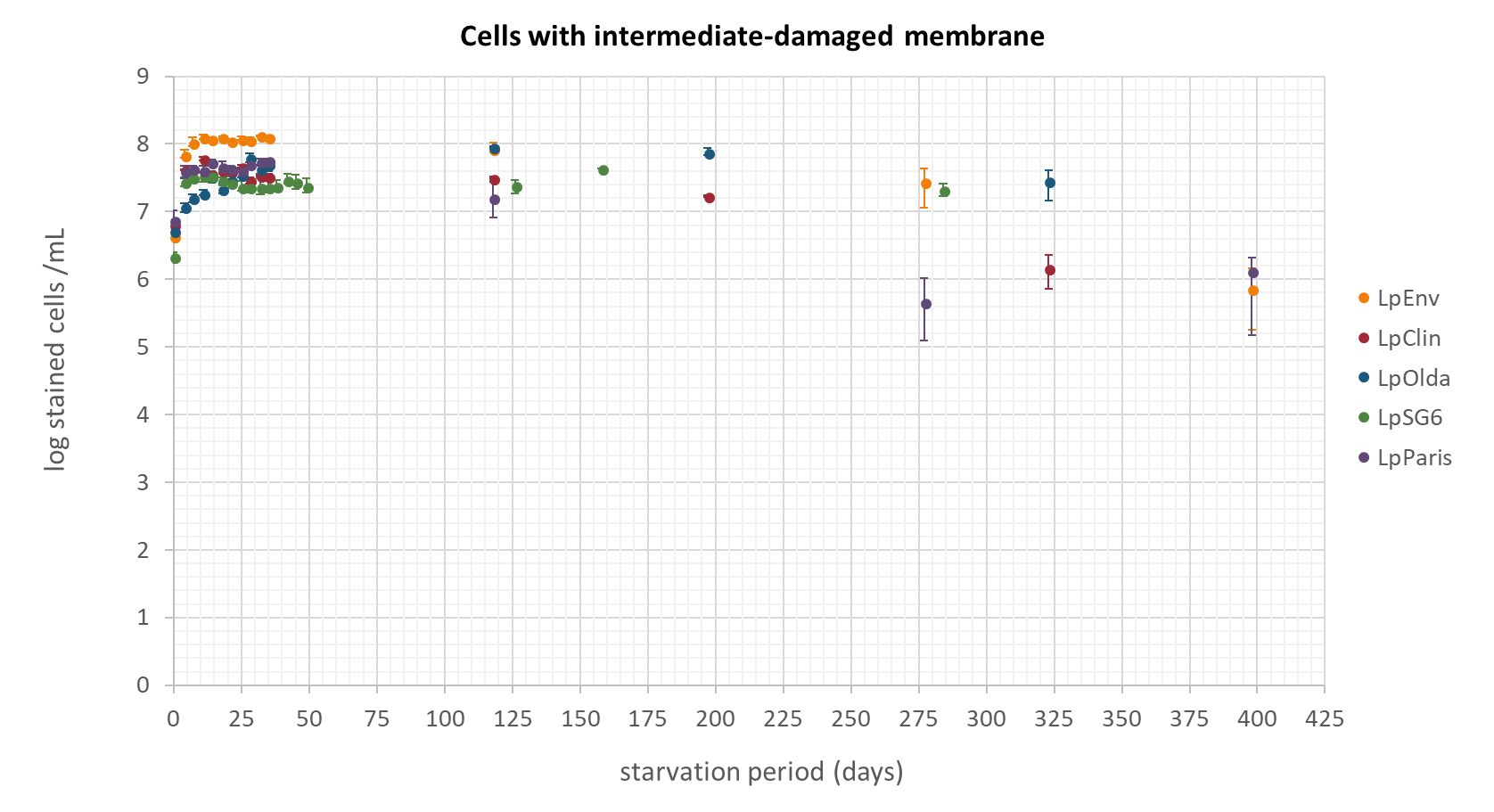 |
| 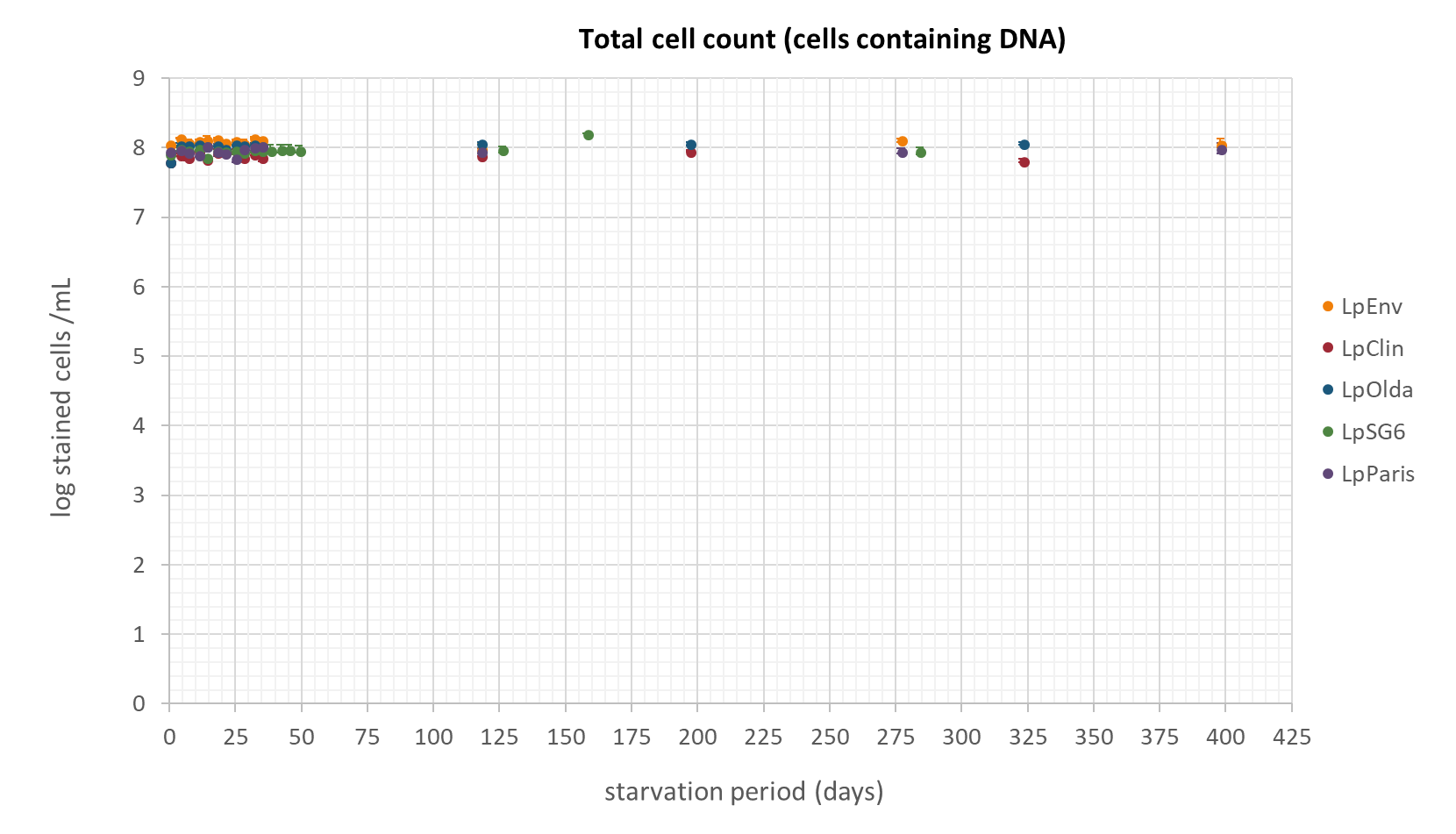 |

E

# Reference

1. Schrammel B, Cervero-Arago S, Dietersdorfer E, Walochnik J, Lück C, Sommer R, et al. Differential development of Legionella sub-populations during short- and long-term starvation. Water Res. 2018;141:417–27.
